# Complete chloroplast genomes of two *Ainsliaea* species and the phylogenetic analysis in the tribe Pertyeae

**DOI:** 10.3389/fgene.2024.1408114

**Published:** 2024-07-23

**Authors:** Xinyu Chen, Yifan Feng, Tianmeng Qu, Hui Chen, Xiaofeng Liu, Liang Pang, Ming Chen, Zhixi Fu

**Affiliations:** ^1^ Key Laboratory of Land Resources Evaluation and Monitoring in Southwest (Sichuan Normal University), Ministry of Education, Chengdu, China; ^2^ College of Life Sciences, Sichuan Normal University, Chengdu, China; ^3^ Wujiao Nature Reserve Administrative Bureau, Jiuzhaigou, China; ^4^ Sichuan Tianshengyuan Environmental Services Co., Ltd., Chengdu, China; ^5^ Sichuan Tianshengyuan Information Technology Co., Ltd., Chengdu, China; ^6^ Sichuan Leshan Ecological Environment Monitoring Center Station, Leshan, China; ^7^ Sustainable Development Research Center of Resources and Environment of Western Sichuan, Sichuan Normal University, Chengdu, China

**Keywords:** *Ainsliaea*, Pertyeae, chloroplast genome, genome comparative analysis, phylogeny

## Abstract

The genus *Ainsliaea* DC. is one of the major groups within the tribe Pertyeae (Asteraceae). It comprises several important Chinese medicinal species. However, the phylogenetic position has undergone a long process of exploration. The complete chloroplast (cp) genome sequences data has not been employed in species identification and phylogeny of *Ainsliaea*. In this study, the complete cp genomes of two *Ainsliaea* species (*A. gracilis* and *A. henryi*) were reported, followed by structural, comparative, and phylogenetic analyses within the tribe Peryteae. Both cp genomes displayed a typical quadripartite circular structure, with the LSC and SSC regions separated by the IR regions. The genomes were 152,959 (*A. gracilis*) and 152,805 (*A. henryi*) base pairs (bp) long, with a GC content of 37.6%. They were highly conserved, containing 134 genes, including 87 protein-coding genes, 37 tRNA genes, 8 rRNA genes, and 2 pseudogenes (*rps19* and *ycf1*). Moreover, thirteen highly polymorphic regions (e.g., *trnK-UUU*, *trnG-UCC*, *trnT-GGU*, *accD-psaI*, and *rpl22-rps19*) were identified, indicating their potential as DNA barcodes. The phylogenetic analysis confirmed the placement of *Ainsliaea* in the tribe Pertyeae, revealing close relationships with the genera *Myripnois* and *Pertya*. In comparison with *Ainsliaea*, *Myripnois* was more closely related to *Pertya*. This study lays a theoretical foundation for future research on species identification, population genetics, resource conservation, and sustainable utilization within *Ainsliaea* and Pertyeae.

## 1 Introduction

The genus *Ainsliaea* DC., a member of the tribe Pertyeae (Asteraceae), predominantly flourishes in the tropics and subtropics of East Asia (Mitsui and Setoguchi, 2012; Zhang et al., 2024), with over fifty species of perennial herbs recorded (Freire, 2007; Mitsui et al., 2008; Gao et al., 2011). In China, the species in the genus *Ainsliaea* demonstrate considerable diversity, with approximately forty distributed, around thirty of them are endemic to the region (Qian, 2000; Wang et al., 2010; Gao et al., 2011; Zhang et al., 2019a; Peng et al., 2020; Zhang et al., 2021). The genus is primarily identified by its basally rosulate and alternate leaves, capitula with few florets, (1-)3(-5), open corollas with deeply irregularly 5-lobed, and pappus of plumose bristles (Freire, 2007; Gao et al., 2011; Zhang et al., 2024). These species thrive in evergreen broad-leaved forests, serving as indicator species for environmental quality. They contribute significantly to ecological maintenance and the conservation of soil and water. Furthermore, *Ainsliaea* encompasses multiple important medicinal plants utilized as traditional Chinese medicines (Qian, 2000; Peng et al., 2020; Zhang et al., 2021) For instance, *Ainsliaea henryi* Diels has historically been employed to treat coughs, asthma, and other diseases (Zeng et al., 2016).

However, the process of determining the phylogenetic position of *Ainsliaea* has undergone a lengthy discovery. Initially, the genus *Ainsliaea* was treated as a member of the tribe Mutisieae (Mutisioideae) based on incomplete morphological studies (Cabrera, 1977; Hind, 2007; Katinas et al., 2008; Gao et al., 2011). Nevertheless, on the basis of cladistic analyses and molecular systematic studies (Kim et al., 2002; Panero and Funk, 2002; Panero and Funk, 2008; Mitsui et al., 2008; Panero, 2008; Freire, 2012), the five closest genera in the tribe Mutisieae, including *Ainsliaea* DC., *Macroclinidium* Maxim. (Japanese endemic), *Catamixis* T. Thomson (Indian endemic), *Myripnois* Bunge, and *Pertya* Sch. Bip. were isolated and collectively constituted a distinct monophyletic taxon, the tribe Pertyeae (Pertyoideae). Furthermore, it was revealed that *Myripnois* exhibited a closer relationship with *Pertya* compared to *Ainsliaea* (Fu et al., 2016). Freire (2017) then proposed the integration of *Myripnois* into the genus *Pertya*. However, this proposal has not obtained widespread acceptance. Therefore, further genomic studies should be conducted to strengthen our understanding of the phylogenetic relationships among *Ainsliaea*, *Pertya*, and *Myripnois.*

A crucial organelle in charge of photosynthesis within green plants is the chloroplast (cp) (Jansen and Ruhlman, 2012). Each chloroplast contains a separate genome from the nuclear genome with lengths of 120–160 kilobase pairs (kp) (Wicke et al., 2011; Daniell et al., 2016). In most angiosperms, a typical cp genome contains a quadripartite circular architecture, consisting of two inverted repeat regions (IRs), a large single-copy region (LSC), and a small single-copy region (SSC) (Palmer, 1985; Bendich, 2004; Mower and Vickrey, 2018). The gradual evolution, peculiar genome structure, and highly conserved sequence are distinguishing features of the cp genomes. Consequently, the analysis of complete cp genome is deemed as the optimal approach for phylogenetic research and the creation of molecular markers within Asteraceae (Ma et al., 2020; Abdullah et al., 2021; Jin et al., 2023). Recently, it has become a valuable tool for offering detailed phylogenetic insights into the evolutionary relationships among various taxa in Asteraceae, including *Artemisia* L. (Jin et al., 2023), *Cavea* W. W. Sm. & J. Small (Yu et al., 2022), *Dolomiaea* DC. (Shen et al., 2020), *Gerbera* L. (Zhang et al., 2019b), *Myripnois* Bunge (Lin et al., 2019), *Nouelia* Franch. (Liu et al., 2022), *Saussurea* DC. (Zhang et al., 2019c; Yun and Kim, 2022), *Sinosenecio* B. Nord. (Peng et al., 2022), and *Taraxacum* F. H. Wigg. (Salih et al., 2017). Nonetheless, only a few complete cp genomes of the tribe Pertyeae have been reported in the NCBI database to date (Lin et al., 2019; Wang et al., 2020; Liu et al., 2021). Additionally, the investigation of phylogenetic relationships among plastomes of *Ainsliaea* and Pertyeae remains unreported.

In this study, the complete cp genomes of two *Ainsliaea* species (*Ainsliaea gracilis* Franch. and *A. henryi*) were obtained and analyzed. The comparative and phylogenetic analyses were subsequently carried out within Pertyeae. The aims of the study were to: (i) illuminate the structure and variation of cp genomes within *Ainsliaea* and Pertyeae; (ii) establish the phylogenetic status of *Ainsliaea* and Pertyeae utilizing cp genomes.

## 2 Materials and methods

### 2.1 Sampling, DNA extraction, and genome sequencing

The samples of *A. gracilis* (*Voucher specimen,* No. DY159) and *A. henryi* (*Voucher specimen,* No. DY133) utilized were collected from Guangwu Mountain, Bazhong City, Sichuan Province, China. The voucher specimens were then deposited at the herbarium of Sichuan Normal University (SCNU), Chengdu City, Sichuan Province, China (contact: Dr. Prof. Zhixi Fu, fuzx2017@sicnu.edu.cn). The extraction of total genomic DNA from fresh leaves was performed following the CTAB DNA extraction protocol (Allen et al., 2006). The quantification and evaluation of the total genomic DNA integrity were assessed using the NanoDrop 2000 Spectrophotometer and Qubit 4 Fluorometer (Thermo Fisher Scientific, Wilmington, DE, USA). DNA libraries were constructed using the Illumina Paired-End DNA Library Kit (Illumina Inc., San Diego, CA, USA) and subsequently sequenced on the NovaSeq 6000 platform with 150 bp paired-end reads (NovoGene Inc., Beijing, China). Eventually, the Illumina Genome Analyzer (Hiseq 2000, Illumina, San Diego, CA, USA) was employed to obtain the raw sequence data. The raw data were then subjected to primary and secondary quality control to yield clean data.

### 2.2 Assembly and annotation of chloroplast genomes

Two sets of clean data, comprising the sequence reads of *A. gracilis* and *A. henryi*, were mapped to the reference sequence of *Ainsliaea latifolia* (D. Don) Sch.-Bip. using Bowtie2 v.2.4.5-Linux (Langmead et al., 2019). Following this, SAMtools v.1.15-Linux (Danecek et al., 2021) was employed to selectively retain only the reads mapped to the reference sequence for subsequent assembly. The cp genomes of two *Ainsliaea* species were assembled by the SPAdes v.3.15.1-Linux with default parameters (Prjibelski et al., 2020). The assembly results were visualized, untangled, inspected, and exported to two complete cp genome sequences using Bandage v.0.9.0-Linux (Wick et al., 2015). Collinearity analysis of the two sequences was performed using MUMmer v4.0.0-Linux (Marcais et al., 2018), with *A. latifolia* as the reference. Subsequently, the obtained sequences were annotated utilizing the Plastid Genome Annotator (PGA) (Qu et al., 2019), referencing the sequences of *Amborella trichopoda* Baill. and *A*. *latifolia*. The annotation results were examined utilizing Geneious R11 (https://www.geneious.com) and adjusted manually as needed. Eventually, the circular map of cp genomes was generated through OrganellarGenomeDRAW (OGDRAW) (Greiner et al., 2019).

### 2.3 Repeat sequence analysis

The simple repeat sequence (SSR) was detected with MISA (Beier et al., 2017). The minimum number of repeats for each nucleotide type was set as follows: 10 repeats for mononucleotide (mono-), 5 repeats for dinucleotide (din-), 4 repeats for trinucleotide (tri-), and 3 repeats for tetranucleotide (tetra-), pentanucleotide (penta-), and hexanucleotide (hexan-). The long repeats were analyzed by REPuter (Kurtz et al., 2001). The analysis encompassed forward (F), reverse (R), complement (C), and palindromic (P) repeats. The maximal repeat size was set at 50 bp, the minimal repeat size was set at 30 bp, and the hamming distance was set at 8.

### 2.4 Comparative genome analysis

The sequences of *A. gracilis* (No. OQ723680), *A. henryi* (No. PP175243), *A. latifolia* (No. MW316662), *Myripnois dioica* Bunge (No. MK784068), *Pertya multiflora* Cai F. Zhang & T. G. Gao (No. MW148616), and *Pertya phylicoides* J. F. Jeffrey. (No. MN935435) were retrieved from GenBank in the NCBI database for comparative cp genome analysis. The contraction and extension of the IR borders within the four major regions (LSC/IRb/SSC/IRa) of the six cp genome sequences were visualized using IRscope (Amiryousefi et al., 2018). The online software mVISTA, with the Shuffle-LAGAN mode (Brudno et al., 2003; Frazer et al., 2004), was employed to compare the plastomes using the sequence of *A. gracilis* as a reference.

### 2.5 Sequence divergence analysis

Python scripts were used to extract the coding regions (CDS) and non-coding regions (IGS) from six sequences for subsequent alignment. These sequences included two from *Ainsliaea* and four from Pertyeae. The sequences were aligned together using the “auto” strategy of MAFFT v.7.475 (Katoh et al., 2019). Nucleotide diversity (Pi) was subsequently calculated using the sliding window of DnaSP v.6.12.03 with window length of 600 bp and step size of 200 bp (Rozas et al., 2017). The Pi values were visualized using an R script.

### 2.6 Phylogenetic analysis

A total of 25 complete cp genomes from Asteraceae family were selected to construct phylogenetic trees to determine the systematic position of *A. gracilis* and *A. henryi*. They represented the major five subfamilies (Asteroideae, Cichorioideae, Gymnarrhenoideae, Pertyoideae, and Carduoideae). *Anthriscus cerefolium* Hoffm. (Apiaceae) and *Kalopanax septemlobus* (Thunb.) Koidz. (Araliaceae) were chosen as outgroups. Two data matrices (complete cp genomes and CDS) were selected for phylogenetic analysis. These 27 sequences data were aligned by MAFFT v.7.475 with default parameters (Katoh et al., 2019), followed by manual inspection and adjustment of sequence differences. The maximum likelihood (ML) and Bayesian inference (BI) trees was inferred with RaxML HPC 2 v8.2.12 (Stamatakis, 2014) and MrBayes v3.2.7a (Ronquist et al., 2012) accessible on CIPRES platform (https://www.phylo.org/), employing the GTRGAMMA substitution model (predicted by ModelTest-NG v0.1.7, Darriba et al., 2020), with 1000 bootstrap replicates (Miller et al., 2010). Finally, the ML and BI trees was visualized and optimized in the program FigTree v.1.4.4 (http://tree.bio.ed.ac.uk/software/figtree).

## 3 Results

### 3.1 Characteristics of the chloroplast genomes

**FIGURE 1:**
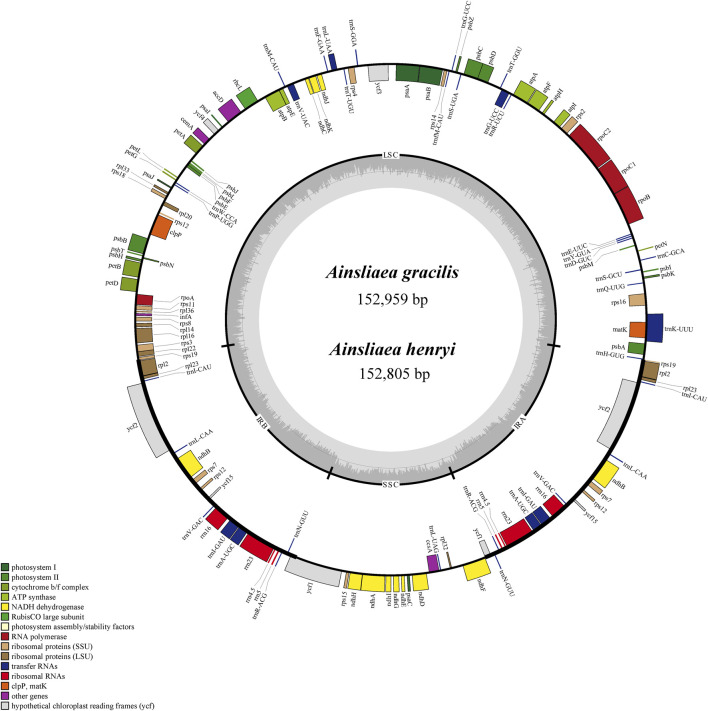
Gene map of the cp genomes of *A. gracilis* and *A. henryi*. Genes located on the inner periphery of the large circle are transcribed clockwise, while those on the outer periphery are transcribed counterclockwise. Genes are color-coded based on respective functions. The dashed region denotes the GC composition of the cp genomes.

The graphical plastome maps of the newly sequenced *Ainsliaea* species (*A. gracilis* and *A. henryi*) are depicted in Figure 1. A total of 134 genes were identified and annotated in the cp genomes, consisting of 87 protein-coding genes, 37 tRNA genes, 8 rRNA, and 2 pseudogenes (Figure 1). Among these genes, 16 contained one intron (*ndhA*, *ndhB*, *petB*, *petD*, *atpF*, *rpl2*, *rps12*, *rpl16*, *rps16*, *rpoC1*, *trnA-UGC*, *trnG-UCC*, *trnI-GAU*, *trnK-UUU*, *trnL-UAA*, *trnV-UAC*) and 2 genes possessed two introns (*clpP* and *ycf3*). It was found to have 19 double-copy genes, including 5 protein-coding genes (*ndhB*, *rpl2*, *rpl23*, *rps7*, *rps12*), 4 rRNA genes (*rrn16*, *rrn23*, *rrn4.5*, *rrn5*), 8 tRNA genes (*trnA-UGC*, *trnG-UCC*, *trnI-CAU*, *trnI-GAU*, *trnL-CAA*, *trnN-GUU*, *trnR-ACG*, *trnV-GAC*), and 2 unknown function genes (*ycf2*, *ycf15*) (Table 2). The gene *ycf1* also has two copies, but one of them was a pseudogene. In addition, the gene *rps12* was a trans-splicing construct. It is noteworthy that the functions of *ycf1*, *ycf2*, *ycf3*, *ycf4*, and *ycf15* are still unknown.

**FIGURE 2:**
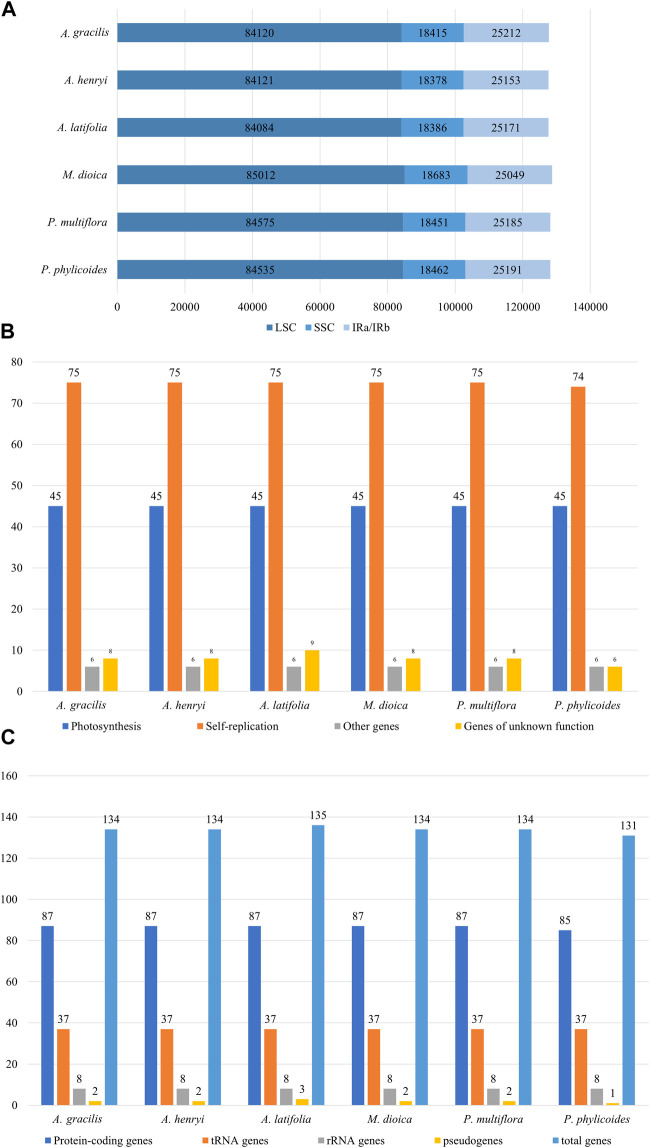
Characteristics of cp genomes in six Pertyeae species. **(A)** Genomes sizes. **(B)** Number of genes. **(C)** Number of genes categorized by functional groups.

The gene structure and content of cp genomes of the two *Ainsliaea* species were highly similar to other four Pertyeae species. The sequence length of the two *Ainsliaea* and four Pertyeae species ranged from 152,805 bp (*A. henryi*) to 153,793 bp (*M. dioica*) (Figure 1; Table 1). These cp genomes exhibited comparable genome structures, comprising 131–135 genes (which include 85–87 protein coding genes, 37 tRNA genes, 8 rRNA genes, and 1-3 pseudogenes, Figure 2 and Table 1). The genes could be categorized into 4 types: photosynthesis-related genes (45), self-replication-related genes (74–75), other genes (6) and genes of unknown function (6–9) (Figure 2). The six genomes contained a pair of IRs (25,049–25,212 bp) separated by the LSC (84,084–85,012 bp) and SSC region (18,378–18,683 bp) (Table 1; Figure 2). The average GC content of the genomes was 37.6%. Compared to the LSC (35.63%–35.83%) and SSC (31.15%–31.31%) regions, the IR regions possessed a higher GC content (43.07%–43.12%)

**TABLE 1:** Characteristics of complete cp genomes of six Pertyeae species.

Genome features	*A. gracilis*	*A. henryi*	*A. latifolia*	*M. dioica*	*P. multiflora*	*P. phylicoides*
Genome size (bp)	152, 959	152, 805	152, 812	153, 793	153, 396	153, 379
LSC size (bp)	84, 120	84, 121	84, 084	85, 012	84, 575	84, 535
SSC size (bp)	18, 415	18, 378	18, 386	18, 683	18, 451	18, 462
IRa/IRb size (bp)	25, 212	25, 153	25, 171	25, 049	25, 185	25, 191
Total GC content (%)	37.6	37.6	37.6	37.6	37.6	37.6
GC content in LSC (%)	35.68	35.70	35.72	35.83	35.63	35.64
GC content in SSC (%)	31.29	31.30	31.31	31.15	31.28	31.20
GC content in IRa/IRb (%)	43.07	43.12	43.11	43.10	43.12	43.12
Number of genes	134	134	135	134	134	131
Protein-coding genes	87	87	87	87	87	85
tRNA genes	37	37	37	37	37	37
rRNA genes	8	8	8	8	8	8
pseudogenes	2	2	3	2	2	1
Accession numbers in GenBank	OQ723680	PP175243	MW316662	MK784068	MW148616	MN935435

### 3.2 Repeat sequence

**FIGURE 3:**
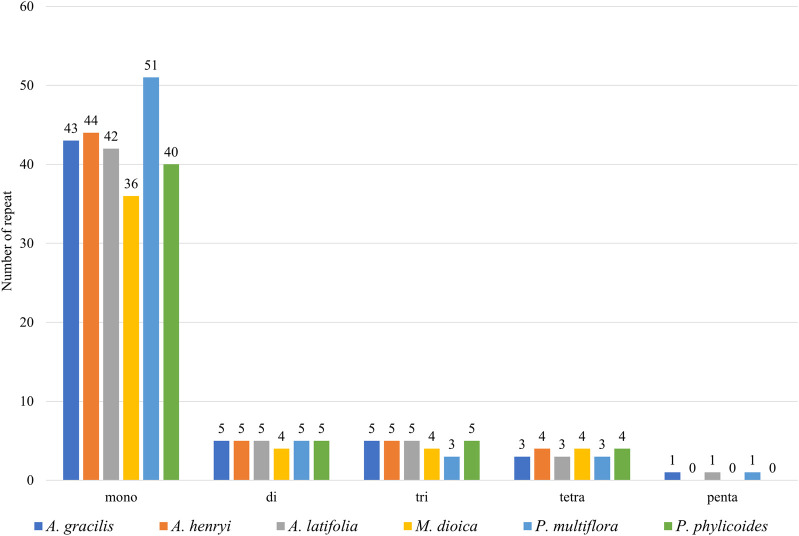
Comparison of the simple sequence repeats (SSRs) across the cp genomes of six Pertyeae species.

**FIGURE 4:**
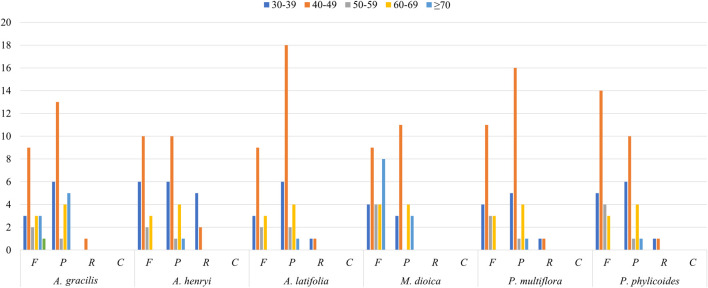
Long repeat sequences in the complete cp genomes of six Pertyeae species categorized as F (forward), P (palindromic), R (reverse), and C (complement).

In total 57, 58, 56, 48, 63, and 54 SSRs were found in *A. gracilis*, *A. henryi*, *A. latifolia*, *M. dioica*, *P. multiflora*, and *P. phylicoides*, respectively (Figure 3). Among these SSRs, mononucleotide repeats were the most abundant, while pentanucleotides repeats were only detected in *A. gracilis* and *P. multiflor*a. Further analysis of the long repeats is provided in Figure 4. The results demonstrated that the number of long repeats in the tribe Pertyeae were highly similar. Palindromes (21–31) were the most prevalent, with a majority of them ranging from 40–49 bp in length. This was followed by forward repeats (17–29) and reverse repeats (1–7), while complement repeats were not observed.

### 3.3 Comparative genome analysis

**FIGURE 5:**
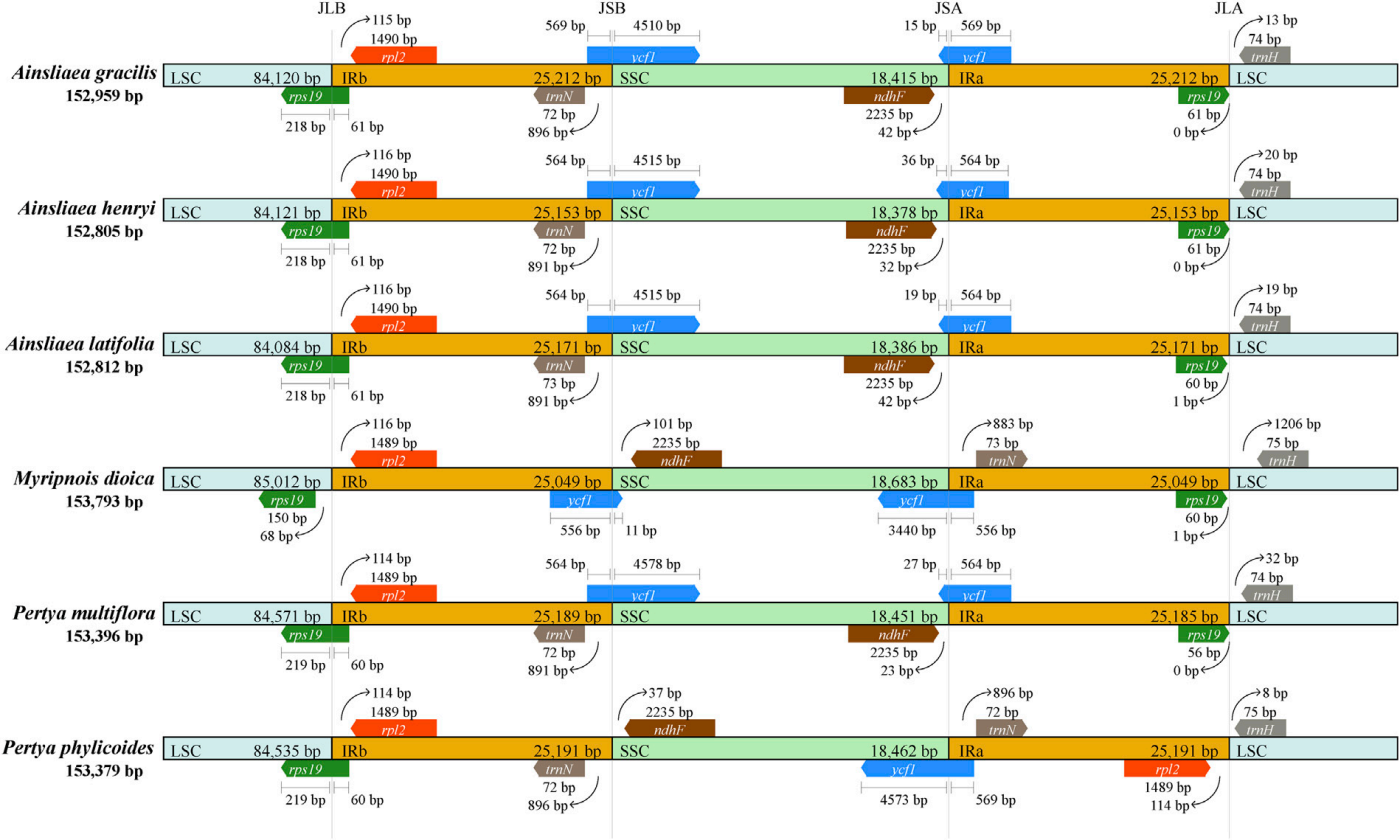
Comparisons of boundaries in the large single copy (LSC), small single copy (SSC), and inverted repeat (IR) regions across cp genomes of six Pertyeae species.

We compared the IR/SC boundaries of the 6 species within Pertyeae (Figure 5). While the lengths of the IR regions were similar among the six species, variations in the extensions and contractions at the IR boundaries were observed. As shown in Figure 5, notably, *A. gracilis* exhibited the longest IR region length (25,212 bp) among the six species. Moreover, substantial differences were observed in the range of each region within Pertyeae. In particular, the *rps19* gene showed expansions ranging from 60 to 61 bp, extending from the LSC to the IRb region in the five species. In *M. dioica*, however, this gene was entirely located within the LSC region, 68 bp away from the LSC/IRb boundary. The *ndhF* gene was found in the SSC regions, both positioned 23–42 bp away from the SSC/IRa boundary, except in *M. dioica* (101 bp away from the IRb/SSC boundary) and *P. phylicoides* (37 bp away from the IRb/SSC boundary). The *ndhF* gene is positioned near the IRa region in the four species and near the IRb region in the other two species. Similarly, the *ycf1* gene was positioned at the IRb/SSC border, except for *M. dioica* and *P. phylicoides*, where it was located at the SSC/IRa border.

**FIGURE 6:**
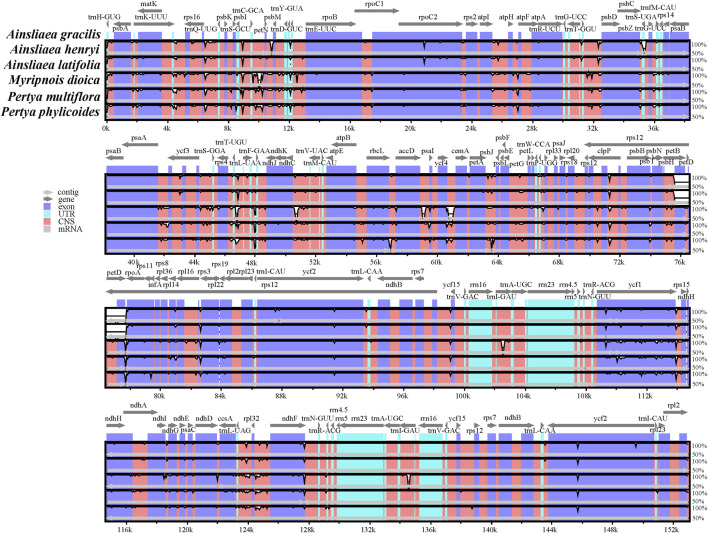
Visualization of genome alignment of cp genomes from six Pertyeae species using *A. gracilis* as the reference by mVISTA. The horizontal axis represents the coordinate in the cp genomes. The vertical scale depicts sequence similarity of aligned regions, with percent identity ranging from 50%–100%.

The complete cp genome sequences of six Pertyeae species were compared using mVISTA online software (Figure 6). While some variation was present, the results indicated a large extent of similarity among these cp genomes. It is worth noting that the SC regions were more variable compared to the IR regions. The conserved non-coding sequences (CNSs) were more diverse than the coding sequences.

### 3.4 Genetic diversity analysis

**FIGURE 7:**
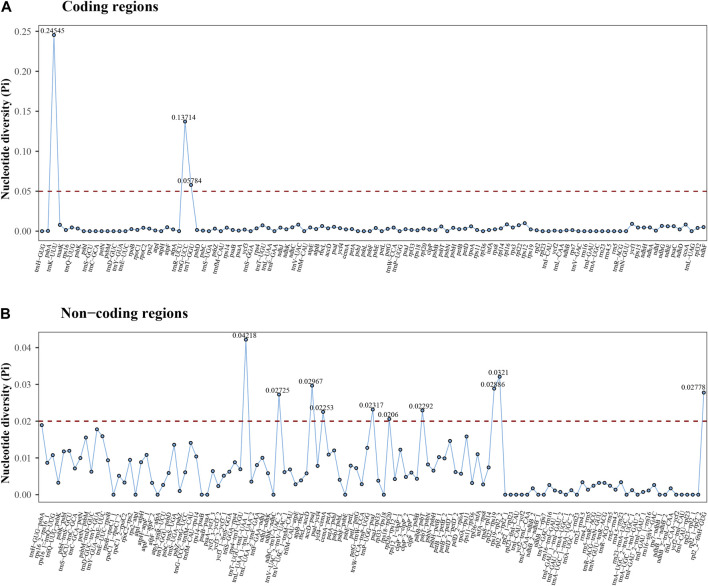
The nucleotide diversity (Pi) values of cp genomes in six Pertyeae species based on **(A)** coding regions and **(B)** non-coding regions. *X*-axis: each coding or non-coding region; *Y*-axis: nucleotide diversity of each region.

Nucleotide diversity (Pi) analysis revealed that a higher proportion of variable sites in non-coding regions on average compared to coding regions (Figure 7). In the coding regions, Pi values ranged from 0 to 0.24545 (*trnK-UUU*), with an average of 0.00633. In the non-coding regions, Pi values ranged from 0 to 0.04218 (*trnT-UGU-trnL-UAA_1*), with an average of 0.00804. Notably, thirteen distinct regions showcased high variability, with Pi values exceeding 0.05 (*trnK-UUU*, *trnG-UCC*, and *trnT-GGU*) and 0.02 (*trnT-UGU-trnL-UAA_1*, *rps19-rpl2_2*, *accD-psaI*, *rpl22-rps19*, *rpl2_2-trnH-GUG*, *ndhC-trnV-UAC_2*, *trnP-UGG-psaJ*, *psbB-psbT*, *ycf4-cemA*, and *rps18-rpl20*). These highly variable regions possess significant potential as informative markers for future phylogenetic analysis at higher taxonomic levels within Pertyeae.

### 3.5 Phylogenetic inference

**FIGURE 8:**
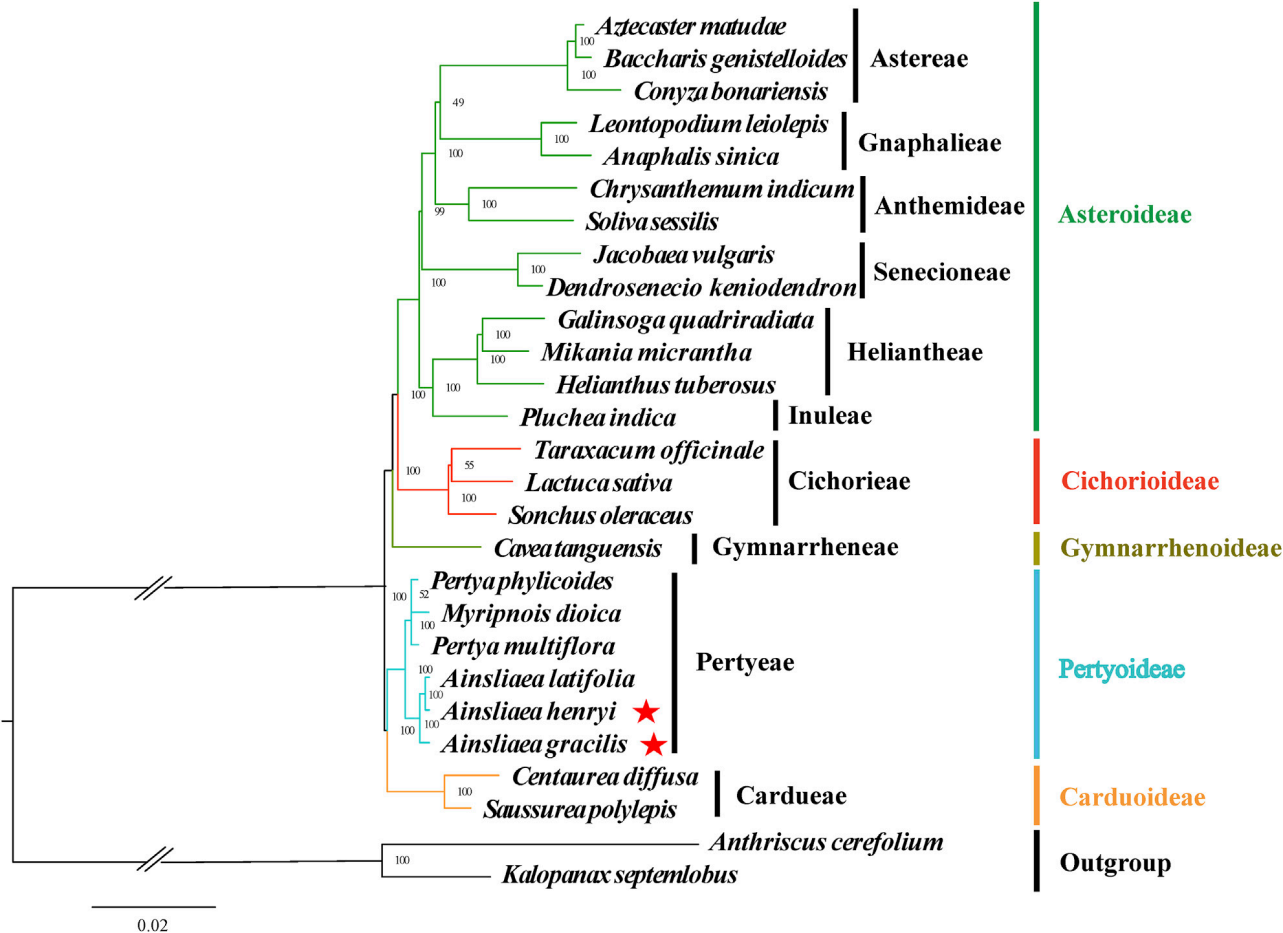
Molecular phylogenetic trees of 25 Asteraceae and 2 outgroups species based on complete cp genomes using maximum likelihood methods. Species are color-coded by subfamily, with branch nodes indicating bootstrap values. The red stars represent the newly sequenced species.

**FIGURE 9:**
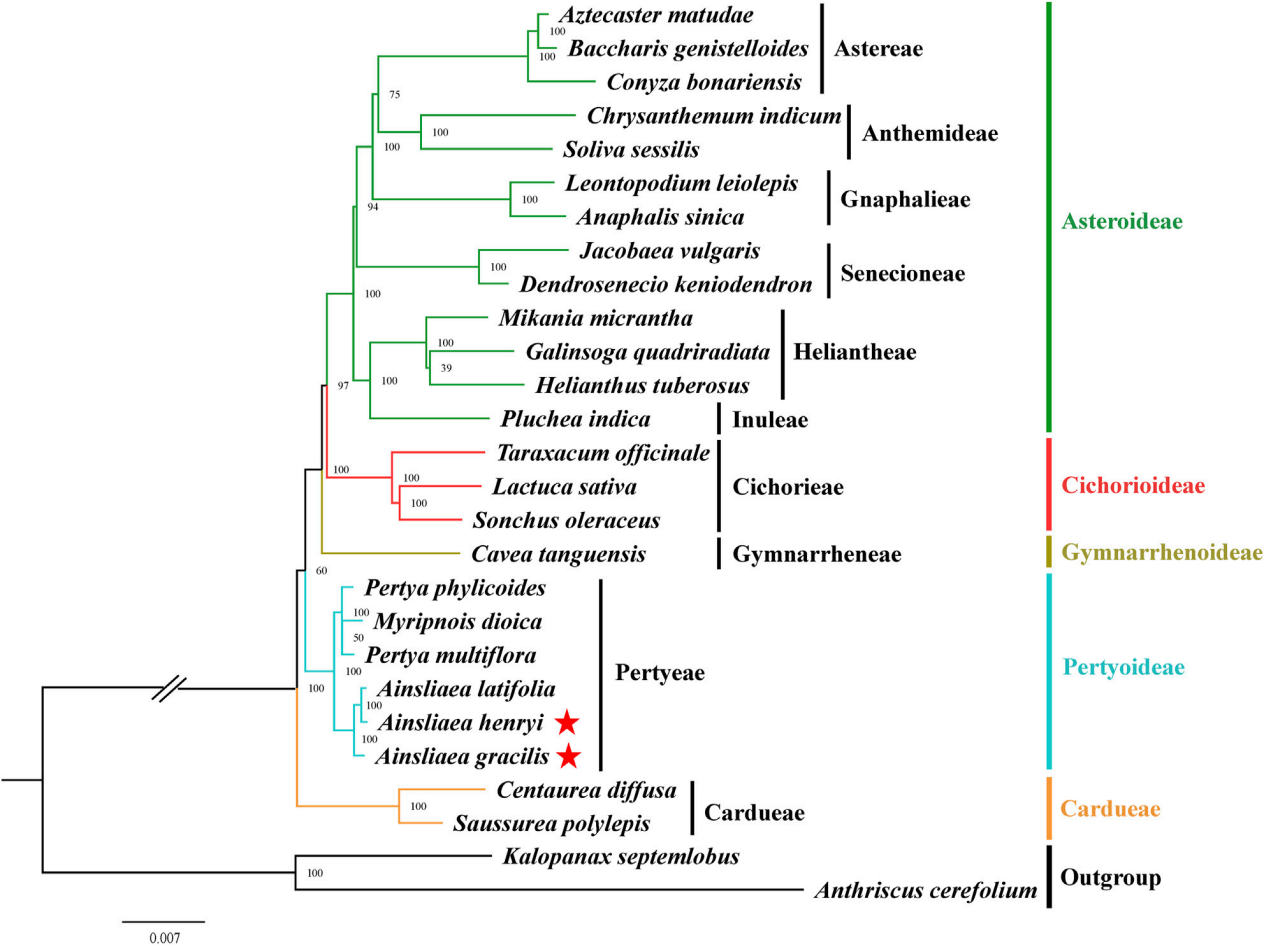
Molecular phylogenetic trees of 25 Asteraceae and 2 outgroups species based on coding DNA sequences using maximum likelihood methods. Species are color-coded by subfamily, with branch nodes indicating bootstrap values. The red stars represent the newly sequenced species.

**FIGURE 10:**
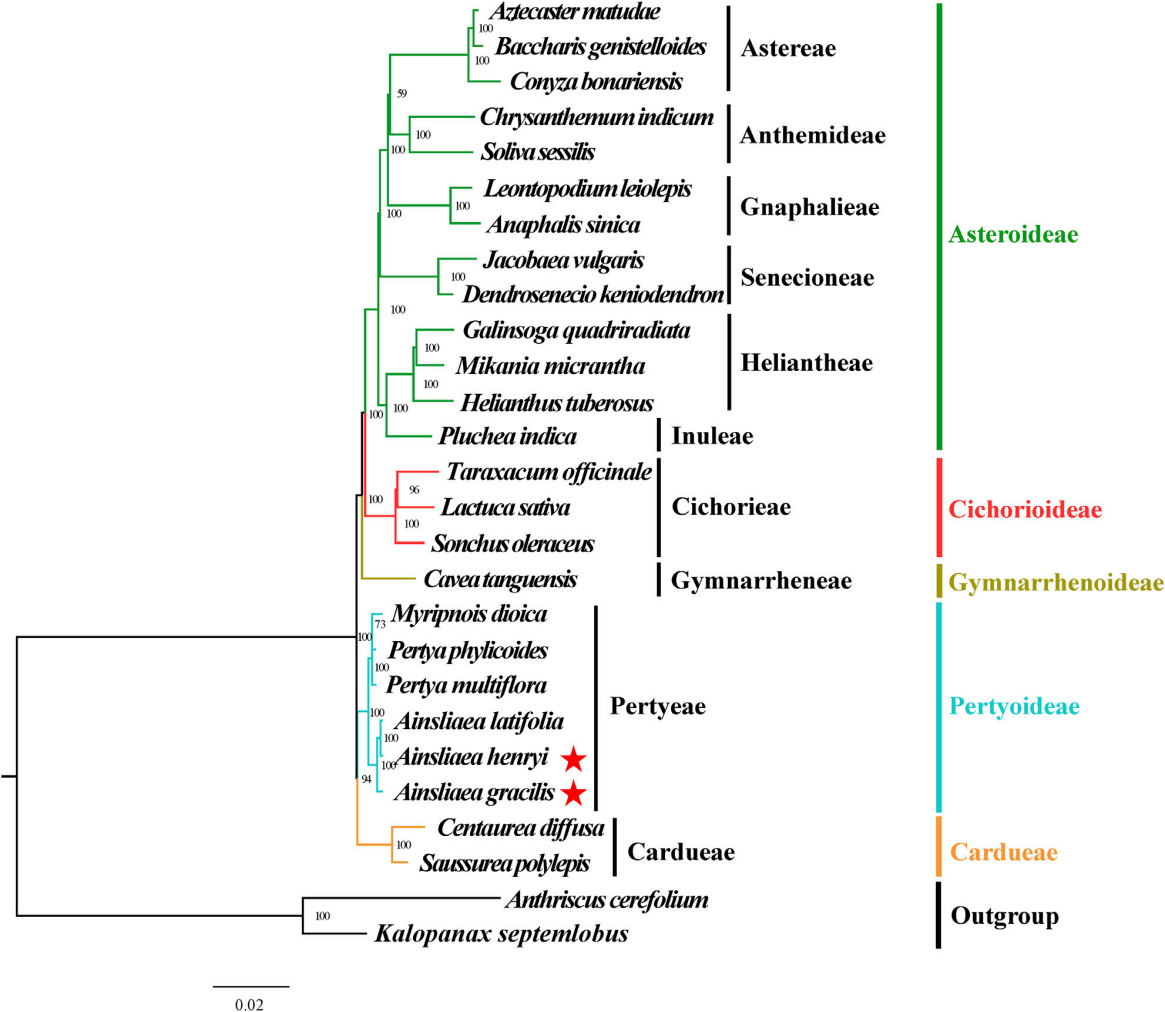
Molecular phylogenetic trees of 25 Asteraceae and 2 outgroups species based on complete cp genomes using Bayesian inference methods. Species are color-coded by subfamily, with branch nodes indicating bootstrap values. The red stars represent the newly sequenced species.

**FIGURE 11:**
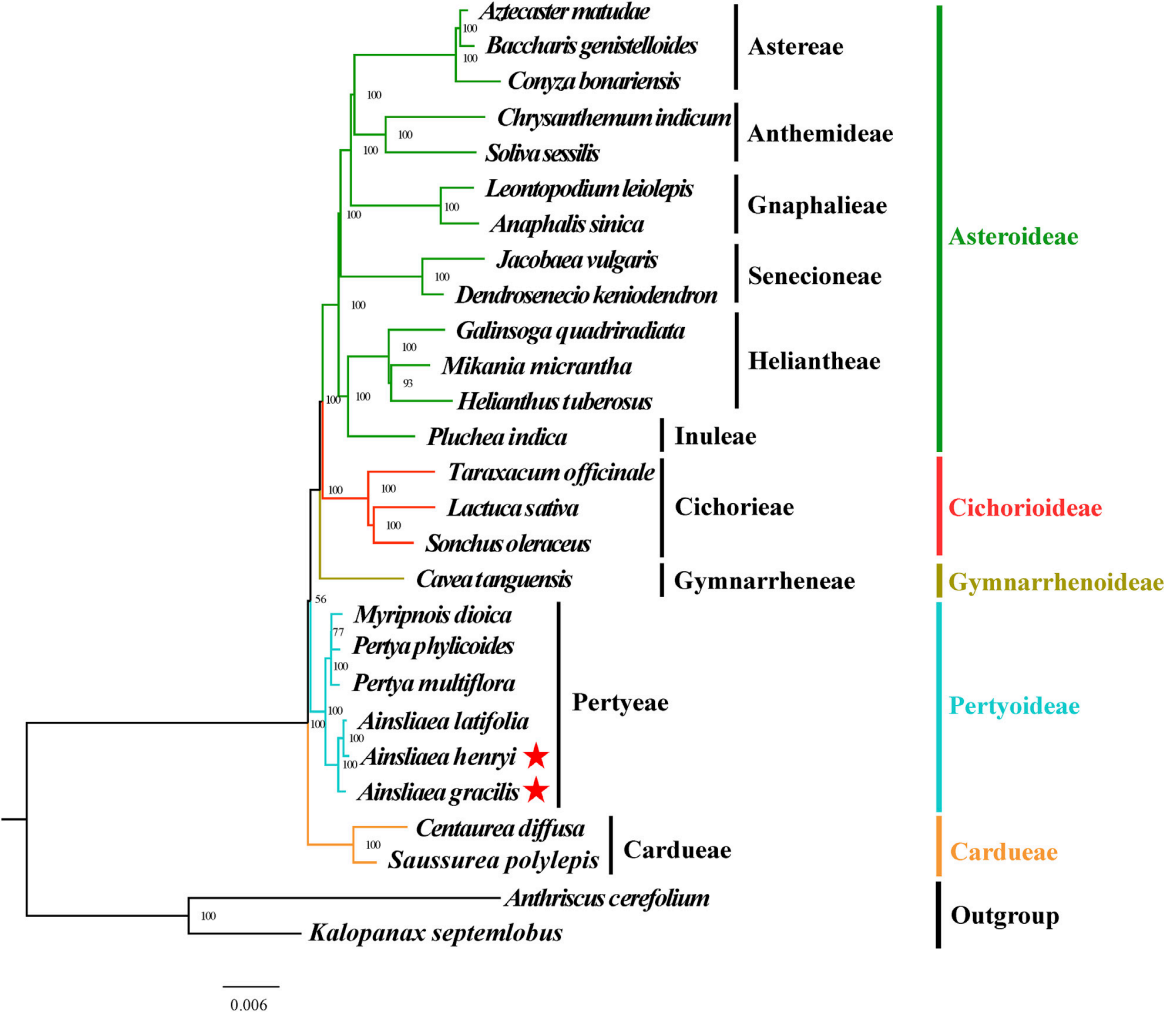
Molecular phylogenetic trees of 25 Asteraceae and 2 outgroups species based on coding DNA sequences using Bayesian inference methods. Species are color-coded by subfamily, with branch nodes indicating bootstrap values. The red stars represent the newly sequenced species.

The ML (Figures 8, 9) and BI phylogenetic trees (Figures 10, 11) were constructed using the complete cp sequences and CDS from 27 species, representing five main clades in Asteraceae. In this analysis, *A. cerefolium* (Apiaceae) and *K. septemlobus* (Araliaceae) were used as outgroups. The phylogenetic relationships inferred from the ML and BI trees based on complete cp genomes (Figures 8, 10) and CDS (Figures 9, 11) were mostly consistent. The phylogenetic trees revealed that all sampled taxa in Asteraceae formed five significant main subfamilies (Asteroideae, Cichorioideae, Gymnarrhenoideae, Pertyoideae, and Carduoideae), encompassing ten tribes (Astereae, Gnaphalieae, Anthemideae, Senecioneae, Heliantheae, Inuleae, Cichorieae, Gymnarrheneae, Pertyeae, and Cardueae). Among these five subfamilies, Pertyoideae was closely allied with Gymnarrhenoideae and Carduoideae. Furthermore, the two newly sequenced species and four additional species from Pertyeae forme a clade (Pertyeae) with robust support. *A. latifolia, A. henryi,* and *A. gracilis* collectively formed a distinct clade, indicating their close evolutionary relationship. Moreover, the clade of *Myripnois* and *Pertya* was closely allied with *Ainsliaea*. Notably, *Myripnois* was identified as nested within the genus *Pertya*. Compared to *Ainsliaea*, *Myripnois* showed a closer relationship to *Pertya*. The results supported that the three genera belonged to the tribe Pertyeae and the subfamily Pertyoideae.

## 4 Discussion

The Pertyeae is a well-represented and widely distributed tribe with abundant species (around 45 spp. endemic) in China (Gao et al., 2011; Fu et al., 2016). The chloroplast, a semi-autonomous genetic organelle, possesses an independent transcription and transport system (Palmer, 1985). In most terrestrial plants, the chloroplast genomes demonstrate highly conserved structures and organization. The genomes typically exist as circular DNA molecules with a size ranging from 120–170 kb (Wicke et al., 2011).

However, only the plastomes of four Pertyeae species have been sequenced to date (Lin et al., 2019; Wang et al., 2020; Liu et al., 2021). Except for *A. latifolia,* the cp genomes of *M. dioica* (Lin et al., 2019), *P. phylicoides* (Wang et al., 2020), and *P. multiflora* (Liu et al., 2021) were individually characterized and employed in conducting separate phylogenetic analysis with other Asteraceae species. In addition, only *M. dioica* was utilized to perform plastome comparative analysis with around 80 species of Asteraceae. Therefore, these four plastomes have not been thoroughly compared. We consequently sequenced and compared the complete chloroplast genomes of *A. gracilis* and *A. henryi* with four related species within Pertyeae tribe.

### 4.1 Genomic characteristics of *Ainsliaea*

The two cp genomes exhibited lengths of 152,959 (*A. gracilis*) and 152,805 (*A. henryi*) bp. The GC content was 37.6% in these cp genomes, as in *Dolomiaea denticulate* (Y. Ling) C. Shih (Shen et al., 2020) and *Sonchus brachyotus* DC. (Wang et al., 2021). These genomes each contained 134 genes, congruent with those of *Sinosenecio albonervius* Y. Liu & Q. E. Yang (Abdullah et al., 2021), which comprised 87 protein-coding genes, 37 tRNA genes, 8 rRNA genes, and 2 pseudogenes (Table 2). Out of 134 genes, 16 contained one intron, while 2 contained two introns. A total of 19 double-copy genes were also detected (Table 2). The detected pseudogenes (*rps19* and *ycf1*) were previously reported in *Ligularia* Cass. (Chen et al., 2018), *Artemisia* L. (Liu et al., 2013) and *Parasenecio* W. W. Sm. & J. Small (Liu et al., 2023). Notably, *rps12* was recognized as a trans-splicing gene, previously reported in other species (Choi and Park, 2015). Moreover, the use of GTG, as a start codon for *rps19*, was reported in other angiosperm cp genomes (Liu and Xue, 2004; Wang et al., 2016).

**TABLE 2:** Genes present in the cp genome of *A. gracilis* and *A. henryi*.

Category	Gene group	Gene name
Photosynthesis	Subunits of photosystem I	*psaA*, *psaB*, *psaC*, *psaI*, *psaJ*
	Subunits of photosystem II	*psbA*, *psbB*, *psbC*, *psbD*, *psbE*, *psbF*, *psbH*, *psbI*, *psbJ*, *psbK*, *psbL*, *psbM*, *psbN*, *psbT*, *psbZ*
	Subunits of NADH dehydrogenase	ndhA*, ndhB*(2), *ndhC*, *ndhD*, *ndhE*, *ndhF*, *ndhG*, *ndhH*, *ndhI*, *ndhJ*, *ndhK*
	Subunits of cytochrome b/f complex	*petA*, petB*, petD*, *petG*, *petL*, *petN*
	Subunits of ATP synthase	*atpA*, *atpB*, *atpE*, atpF*, *atpH*, *atpI*
	Large subunit of rubisco	*rbcL*
Self-replication	Proteins of large ribosomal subunit	rpl2*(2), *rpl14*, rpl16*, *rpl20*, *rpl22*, *rpl23(2)*, *rpl32*, *rpl33*, *rpl36*
	Proteins of small ribosomal subunit	*rps11*, rps12*(2), *rps14*, *rps15*, rps16*, *rps18*, *rps19*, *#rps19*, *rps2*, *rps3*, *rps4*, *rps7(2)*, *rps8*
	Ribosomal RNAs	*rrn4.5(2)*, *rrn5(2)*, *rrn16(2)*, *rrn23(2)*
	Subunits of RNA polymerase	*rpoA*, *rpoB*, rpoC1*, *rpoC2*
	Transfer RNAs	trnA-UGC*(2), *trnC-GCA*, *trnD-GUC*, *trnE-UUC*, *trnfM-CAU*, *trnF-GAA*, trnG-UCC*(2), *trnH-GUG*, *trnI-CAU(2)*, trnI-GAU*(2), trnK-UUU*, *trnL-CAA(2)*, trnL-UAA*, *trnL-UAG*, *trnM-CAU*, *trnN-GUU(2)*, *trnP-UGG*, *trnQ-UUG*, *trnR-ACG(2)*, *trnR-UCU*, *trnS-GCU*, *trnS-GGA*, *trnS-UGA*, *trnT-GGU*, *trnT-UGU*, *trnV-GAC(2)*, trnV-UAC*, *trnW-CCA*, *trnY-GUA*
Other genes	Acetyl-CoA carboxylase	*accD*
	c-type cytochrome synthesis gene	*ccsA*
	Envelope membrane protein	*cemA*
	Maturase	*matK*
	Protease	clpP**
	Translation initiation factor	*infA*
Genes of unknown function	Conserved hypothetical chloroplast ORF	*#ycf1*, *ycf1*, *ycf2(2)*, ycf3**, *ycf4*, *ycf15(2)*

Notes: Gene*/**: Gene with one/two introns; *#Gene*: Pseudogene; *Gene(2)*: Double-copy genes.

### 4.2 Genomic comparison of Pertyeae species

The cp genomes of six Pertyeae species revealed significant conservation in size, gene content, structure, and other characteristics. A typical quadripartite circular structure was present in the genomes, with a distinct separation between the LSC and SSC regions by the IR regions. It was accordant with the cp genomes observed in other Pertyeae species (Lin et al., 2019; Wang et al., 2020; Liu et al., 2021). However, the cp genome size of the genus *Ainsliaea* (152,805–152,959 bp) is relatively shorter compared to the genera *Pertya* and *Myripnois* (153,379–153,793 bp). According to analysis of the cp genome divergence, *Ainsliaea* appears to possess low levels of sequence divergence and generally conserved plastomes. The intergenic spacers were identified as the most divergent regions, with non-coding regions showing greater divergence than coding regions. Previous multispecies investigations (Hu et al., 2020) had demonstrated that intergenic spacers were highly informative phylogenetic markers.

### 4.3 Sequence repeats analysis

SSRs, composed of 1-6 nucleotide repeat units, are prevalent across cp genomes. Previous studies have employed them for species identification, genetic diversity, and polymorphism research (Deguilloux et al., 2004; Redwan et al., 2015). A total of 336 SSRs were identified in the cp genomes of Pertyeae. Mononucleotide repeats predominated in sequences. This phenomenon might be caused by the fact that SSRs commonly consist of polyA or polyT repeats (Zhang et al., 2019d). It was congruent with the cp genomes of most Asteraceae species (Choi and Park, 2015; Abdullah et al., 2021; Liu et al., 2023). The discovery of these newly identified SSRs will present valuable resources for future development of genetic markers for *Ainsliaea* species. Moreover, this study identified thirteen highly polymorphic loci (e.g., *trnK-UUU*, *trnG-UCC*, *trnT-GGU*, *accD-psaI*, and *rpl22-rps19*). In the tribe Pertyeae, these variable regions could serve as possible DNA barcodes for species identification and phylogenetic analysis.

### 4.4 Phylogenetic analysis

The Asteraceae, recognized as one of two largest and most diverse families of blooming plants worldwide (Bremer and Anderberg, 1994; Funk et al., 2005; Anderberg et al., 2007), comprises sixteen subfamilies (Susanna et al., 2020; Zhang et al., 2024). In our study, 5 of the 16 subfamilies were sampled. Most nodes in the phylogenetic trees displayed high support values and were similar. The phylogenetic relationships of five subfamilies were consistent with earlier investigations (Funk et al., 2009; Fu et al., 2016; Panero and Crozier, 2016; Mandel et al., 2017). Previous studies have indicated the phylogenetic position and demographic histories of the tribe Pertyeae utilizing short DNA fragments (e.g., *ndhF*, *rbcL*, and *matK*) or the complete cp genomes data. For instance, Zhang (2024) reconstructed an updated phylogeny of *Ainsliaea* based on the plastid *ndhF* and nrDNA (ITS, ETS) sequences. Mitsui and Setoguchi (2012) addressed the demographic histories of adaptively diverged riparian and non-riparian species of *Ainsliaea* using 10 nuclear DNA loci (e.g., CHS, GTF). Fu et al. (2016) proposed that Pertyeae (as recognized by Panero and Funk, 2002) was sister to the tribes Cardueae and Gymnarrheneae and nested above the subfamily Carduoideae. Moreover, Pertyeae also has been suggested as a sister group to the tribes Cichorieae (Lin et al., 2019) and Cardueae (Wang et al., 2020). However, research on the cp genomes of *Ainsliaea* has not yet been conducted to date.

The phylogenetic analysis elucidated the taxonomy placement of the genus *Ainsliaea* within the tribe Pertyeae (Pertyoideae). Compared to *A. gracilis*, *A. henryi* and *A. latifolia* exhibited the closest phylogenetic relationship and clustered together, which is consistent with the findings of Zhang (2024). We also identified that the genus *Ainsliaea* was closely related to *Myripnois* and *Pertya*. This finding was accordant with previous studies (Kim et al., 2002; Fu et al., 2016). Based on previous morphological studies, *Myripnois* was delineated as a distinct genus (Gao et al., 2011). However, phylogenetic analysis confirmed that *Myripnois* was nested within *Pertya* in our study, aligning with the results derived from cladistic analysis (Freire, 2017).

## 5 Conclusion

The complete chloroplast genomes of two *Ainsliaea* species were sequenced, characterized, and analyzed. Phylogenetic analysis confirmed previous studies by placing the genera *Ainsliaea*, *Pertya*, and *Myripnois* within the tribe Pertyeae (Pertyoideae). Additionally, it confirmed that *Myripnois* was found to be nested within *Pertya*, indicating a closer relationship to *Pertya* rather than to *Ainsliaea*. The phylogenetic results in this study aligned with previous findings and suggested a potential reevaluation and refinement of taxonomic classifications and phylogenetic relationships within the tribe Pertyeae.

The plastomes of two *Ainsliaea* species demonstrated a typical quadripartite structure, closely resembling those of other Pertyeae species in terms of genomic size, structure, and gene content. Despite overall conservation, the cp genomes of six Pertyeae species presented some degree of variations. Thirteen highly polymorphic regions located at coding regions and non-coding regions (e.g., *trnK-UUU*, *trnT-GGU*, *accD-psaI*, and *rpl22-rps19*) offered significant potential for developing DNA barcodes. These regions could greatly enhance species identification within tribe Pertyeae. The valuable insights of this study will improve our comprehension of cp genomic data and lay a foundation for phylogenetic relationship of the genus *Ainsliaea* and tribe Pertyeae.

## Data Availability

The datasets presented in this study can be found in online repositories. The names of the repository/repositories and accession number(s) can be found below: https://www.ncbi.nlm.nih.gov/genbank/, OQ723680; https://www.ncbi.nlm.nih.gov/genbank/, PP175243; https://www.ncbi.nlm.nih.gov/genbank/, MW316662; https://www.ncbi.nlm.nih.gov/genbank/, MK784068; https://www.ncbi.nlm.nih.gov/genbank/, MW148616; https://www.ncbi.nlm.nih.gov/genbank/, MN935435.
